# The Use of a Water Soluble Flexible Substrate to Embed Electronics in Additively Manufactured Objects: From Tattoo to Water Transfer Printed Electronics

**DOI:** 10.3390/mi9090474

**Published:** 2018-09-17

**Authors:** Brice Le Borgne, Emmanuel Jacques, Maxime Harnois

**Affiliations:** 1 Advanced Technology Institute, Electrical and Electronic Engineering, University of Surrey, Guildford GU2 7XH, UK; b.h.leborgne@surrey.ac.uk; 2 Département Microélectronique & Microcapteurs, UMR CNRS 6164, Institut d'Électronique et des Télécommunications de Rennes, Université Rennes 1, Campus de Beaulieu, 35042 Rennes CEDEX, France; emmanuel.jacques@univ-rennes1.fr

**Keywords:** water soluble substrates, water transfer printing, additive manufacturing, 3D electronics, flexible electronics, stretchable electronics

## Abstract

The integration of electronics into the process flow of the additive manufacturing of 3D objects is demonstrated using water soluble films as a temporary flexible substrate. Three process variants are detailed to evaluate their capabilities to meet the additive manufacturing requirements. One of them, called water transfer printing, shows the best ability to fabricate electronics onto 3D additively manufactured objects. Moreover, a curved capacitive touchpad hidden by color films is successfully transferred onto the 3D objects, showing a potential application of this technology to fabricate fully additively manufactured discrete or even hidden electronic devices.

## 1. Introduction

Additive manufacturing (AM) is a relatively new process that has been called the “third industrial revolution” [1,2,3]. Rapid prototyping has benefited from the versatility of additive manufacturing and structural parts are now available off the shelf [4,5]. In the context of the Internet of Things (IoT), embedding smart devices, such as conformal electronics (e.g., antennas, sensors, interconnects, electronic circuits...) to structural parts will be one of the next technological steps of additive manufacturing technology [6,7,8,9]. Consequently, literature is replete with many works focusing on novel materials and methods that can solve such challenging issues. Indeed, additive manufacturing is a good candidate to allow the fabrication of highly conformal electronics directly onto 3D daily-life objects [7,8,10]. It could be directly integrated into the manufacturing sequence of the fabrication process of new “smart parts”. Two main approaches have been developed and can be summarized as follows.

The first approach consists of the integration of electronics directly on the 3D objects. In this case, the structural part can be fabricated using a conventional 3D printing technique such as fused deposition modeling (FDM) or stereolithography (SLA) [11]. The electronics can be fabricated using 3D printing technologies such as inkjet printing [12], aerosol jetting (AJ) [13,14], or laser direct structuring (LDS) [9]. Note that, concerning AJ or LDS techniques, complex and expensive five-axis displacements are required to enable the integration of electronics on all the faces of the object’s structural part [15], For LDS, the laser can be expensive and require important health and safety controls as its use can harm operators. Moreover, all the surfaces where conductive paths are needed must be accessible to: (i) the laser beam (for LDS) for activation or (ii) conductive ink stream for AJ processing, which constitutes a major limitation of the technologies. Moreover, to date, the pattern size resolution and available materials are not compatible with the fabrication of integrated electronic components (millimetric scale rather than micrometric scale).

Another promising approach consists of the fabrication of electronics on flexible or stretchable planar substrates before the transfer step onto the 3D object [16,17,18,19,20,21]. This approach benefiting from the advantages of subtractive cleanroom processing that allows the use of a micrometric accuracy and a wide range of compatible materials (insulators, conductors, and semiconductors). However, such a technology requires the flexible substrate to remain at the end of the process, which limits the span of application. For these reasons, innovative new fabrication methods are under development. One of them consists of the fabrication of electronics onto a water soluble substrate that can be easily dissolved in order to transfer only the electronics wrapping a 3D shape. Such technologies have been called electronics tattoo, decal electronics [22,23,24,25,26], and more recently, water transfer printed electronics (WTP) [27,28].

In this work, we investigate the capability of the aforementioned techniques to be integrated into the process flow of additive manufacturing technology. In the first section, three process variants are studied. In the second part, the fabrication and characterisation of a capacitive touchpad illustrate the capabilities of the most promising technology (the WTP) based on the requirements of the additive manufacturing process [27]. Moreover, experiments performed in the last section highlight that water soluble film used as a temporary flexible substrate can be a promising candidate to fabricate a fully additively manufactured conformal hidden electronics device.

## 2. Materials and Methods

### 2.1. Characterization

Top-view images and films were obtained by PENTAX K70D equipped with a ZOOM macro 50 mm (Pentax, Tokyo, Japan). Electrical measurements were performed using a probe station, a Keithley 2636A, and Labview. Surface mounted devices were connected to the aluminum ends by conductive epoxy (reference CW2400; Chemtronics, Glenview, IL, USA). 

### 2.2. PVA Processing

A solution of poly-vinyl alcohol (PVA; Mw 9000–10,000, 80% hydrolyzed from Aldrich, Saint-Louis, MO, USA) was prepared by mixing DI water and PVA powder (5:1 ***w***/***w*** water/PVA). The 0.4 µm filtered solution of PVA was spin-coated to obtain a 50 µm layer thickness and then baked at 100 °C for 2 h to dry the film. PVA spin-coating was performed at a low rotation velocity and acceleration (velocity = 20 rpm and acceleration = 10 rpm·s^−1^) for good thickness uniformity. Indeed, baking spin-coated PVA may lead to the non-uniform thickness, mainly due to evaporative flow (coffee ring effect). As described in a previous study, when PVA film is obtained by spin coating, an under-layer of photoresist can be deposited in order to facilitate the release of PVA at the end of the process (before the transfer step). The processing above is appropriate for small-area electronics. For large-area applications that require inkjet printing technology, for instance, the commercial product of PVA laminated on Polyethylene terephthalate (PET, solublon—PET 75 µm thick and PVA 30 µm thick, Aicello, Nagoya, Japan) films is more suitable. 

### 2.3. Thin Film Patterning

Thin film patterning: Water Transfer Printing technology requires that each thin film layer of the devices must be processed on a water-soluble substrate. Many routes to solve this issue have already been proposed. 

Inkjet printing of silver ink: All the printing experiments were performed using a CERADROP^©^ X-series printer (Limoges, France). Inks were used without filtering steps.

ANP 40LT15C silver ink from Advanced Nano Products Co., Ltd. (Seoul, Korea) has been used for all the printing experiments. Firing voltage and printing frequency have been adjusted to respect the stable jetting criterion (no satellite droplets, straight jetting…) [29,30,31]. Silver ink has been printed using a Fujifilm printhead (256 nozzles, Tokyo, Japan) and baked in a convection oven at a temperature of 120 °C for 1 h. Epoxy-based ink (Su8-2000 series; MicroChem, Westborough, MA, USA) has been printed using a 16-nozzle cartridge (Dimatix^©^, Tokyo, Japan) employing the experimental parameters already reported, baked at 95 °C for 5 min in an oven followed by UV (λ = 365 nm) exposure, and baked again in an oven at 95 °C for 5 min.

Thermally evaporated metal: a 150 nm thick aluminum thin film can also be thermally evaporated using homemade equipment. The shadow mask or reactive ion etching (RIE) method has been used to pattern the aluminum layer. In the case of RIE, chlorinated gas was used in ICP/RIE (inductive coupled plasma/reactive ion etching) equipment to etch aluminum (working pressure: 5 mT; plasma power: 100 W; flow rate: 30 sccm) after the classical lithographic process using S1818 photoresist (MicroChem, Westborough, MA, USA).

Dipping and transfer steps: PVA film was placed on the water surface and dissolved before dipping the object through the floating pattern. The object was withdrawn and dried. Van der Waals force allowed the pattern to stick to the 3D object.

### 2.4. Additive Manufacturing (the Structural Part)

All the FDM experiments have been performed using a commercial printer (Dagoma NEVA, Dagoma, France). The filament made of PLA (Polyactic Acid) with a diameter of 1.75 mm has been deposited at a melting temperature of 215 °C. The reference of the filament is dailyfil.

### 2.5. Water Transfer Printing Setup

A homemade robot has been used to perform the experiments dealing with water transfer printing technology. It consisted of X, Y, and Z motorized axes. A transparent dip tank (30 × 30 × 35 cm) was used, allowing the acquisition of films of the process and alignment of the floating pattern and the 3D objects before the dipping step. Note that rigid guides that are made of stiff metal plates were fixed on the side of the dip tank. The space between the plates could be adjusted to fit the size of the PVA substrate. Such additional guides are required in order to avoid the spreading of patterns when the PVA substrate is fully dissolved. Note that the dipping speed was fixed to 1.5 mm s^−1^.

## 3. Results

### 3.1. PVA Processing Variants

#### 3.1.1. Electronics Tattoo Capabilities (Process Variant 1)

The concept of electronics tattoo was introduced more than five years ago, mainly for medical applications [24]. The concept relies on the fabrication of electronics onto a water-soluble substrate. It is worth noting that PVA (polyvinyl alcohol) or silk are the most frequently reported substrate materials [32]. As the substrate is water soluble, most of the time the electronics are not directly fabricated on it, but transferred from a temporary carrier substrate. Recently, new routes have been reported to fabricate electronics directly on the substrate, suppressing any additional transfer step [27]. The second step of electronics tattoo relies on the transfer of electronics onto the 3D object. In this case, electronics face the object and the hydrosoluble substrate is partially dissolved in water. In this work, this concept is used and adapted to the field of additive manufacturing in order to evaluate how it could be integrated in a process flow.

**Figure 1:**
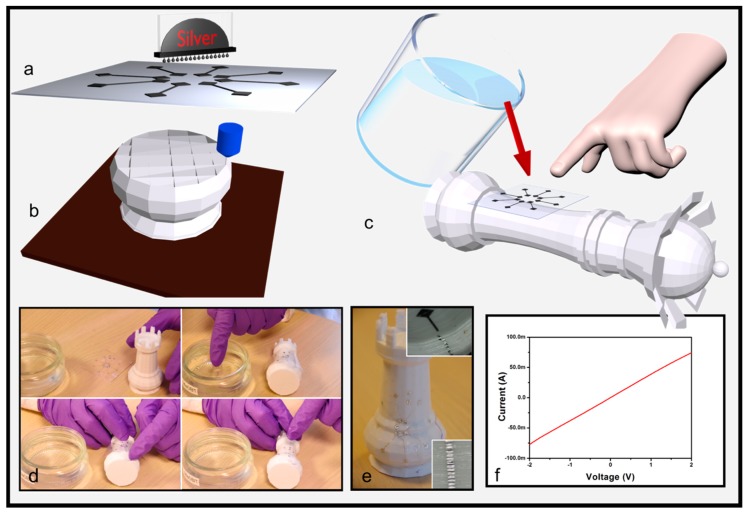
Electronics tattoo technology used to transfer interconnects onto object additively manufactured. Illustrations showing: (**a**) interconnects printed on water soluble substrate (poly-vinyl alcohol, PVA); (**b**) daily-life 3D object additively fabricated using fused deposition modeling (FDM); (**c**) water is applied on PVA substrate. The dissolution of PVA films helps to conformally wrap the 3D object with interconnects. Optical pictures showing: (**d**) the process; (**e**) a chess piece decorated with metallic ribbons arranged in star configurations. The bottom right inset shows ribbons conformally wrapped onto microscopically wave shaped surface (100 µm amplitude). The top right inset shows that when ribbons are transferred onto macroscopic sharp edges, conformal wrapping cannot be obtained. (**f**) I(V) curve.

Figure 1 shows the electronics tattoo process. In this process, an FDM printer is used to provide the structural part of the 3D object and metallic interconnects are patterned to functionalize the object (i.e., silver using inkjet printing or thermally evaporated aluminum). The 3D scheme and optical pictures of Figure 1a, in addition to Figure S1, show the first step of the process. Interconnects can be patterned onto the PVA substrate using inkjet printing (Figure S1a) or photolithography (Figure S1b). The materials and patterning methods of the metal layer can be chosen according to the processes needs. Typically, lithography is employed for small-area processing at high accuracy or printed electronics for large-area processing at a medium accuracy. In this work, the details of the material and patterning method are described in the experimental section. In parallel to the fabrication of electronics, the structural part can be fabricated using FDM, as shown in the Figure 1b. The flexible PVA substrate is then placed facing the 3D object and water is poured to partially dissolve the substrate, as shown in Figure 1c and in the Supplementary Video S1. Optical pictures in Figure 1d highlight the method employed to stick the PVA film to the 3D object. Note that, for all the experiments performed in this work, the design of the interconnects did not change. The metallic ribbons are designed in a star shape, 150 nm thick, 15 mm long, and 150 µm wide, with two squared ends of 2 × 2 mm. As reported in other works dealing with electronics tattoo, a finger is firstly dipped into water and then pressed onto the PVA to dissolve it. Consequently, as expected, conformal wrapping is observed when the topology does not display sharp edges, as shown in the top right inset of Figure 1e. However, as highlighted in the bottom right inset of Figure 1e, when the 3D object shows sharp edges (bending angle value approximately equals 110°), interconnects break. These observations highlight that electronics tattoo is a convenient method to wrap objects displaying a low macroscopic roughness, even if the local microscopic roughness is high, as already observed in the work of Rogers. Furthermore, the electrical characteristics in Figure 1f show an ohmic behavior when ribbons are transferred onto the curved surface of the object. However, the same behavior cannot be observed when the ribbons are transferred onto a sharp edge. Consequently, when large-sized objects showing sharp edges have to be wrapped, the electronics tattoo is not adapted. Consequently, such technology does not fit the requirements of additive manufacturing technology. Thus, others techniques have to be developed to overcome this issue.

#### 3.1.2. 3D Object Additively Manufactured on PVA (Process Variant 2)

**Figure 2:**
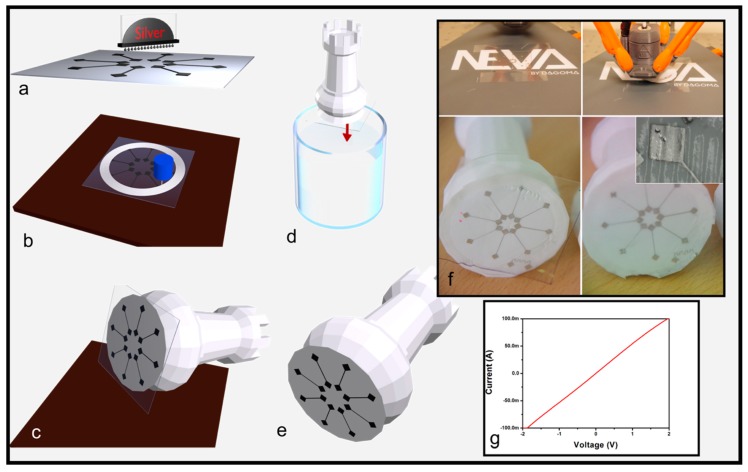
Process variant used to transfer interconnects onto 3D objects additively manufactured. 3D schemes showing: (**a**) interconnects printed on water soluble substrate; (**b**) the PVA substrate is attached on the plate of the FDM equipment and the object is fabricated on top of the interconnects; (**c**) 3D object is detached from the plate; (**d**) the 3D object is dipped into a water bath; (**e**) PVA is dissolved and interconnects are conformally attached to the 3D object; (**f**) optical images showing: (i) In the first row, the PVA substrate attached to the plate (left side) and the FDM printing onto the PVA substrate (right side); (ii) in the second row, the bottom face of the 3D object after detachment of the plate but before the PVA wet etching (left side) and the final results after PVA wet etching (right side); the inset shows a zoom on a branch of the star showing conformal wrapping of the metallic patterns without mechanical failure; (**g**) I(V) curve.

Figure 2 shows a variant of the electronics tattoo process adapted to fit the requirement of the AM process. Here, FDM additive manufacturing technology is used to provide the structural part of the object and metallic interconnects are separately fabricated to functionalize the object. Figure 2a shows the first step of the process. As for the first process, the interconnects can be patterned onto the PVA flexible substrate using inkjet printing (Figure S1a) or photolithography (Figure S1b). In the second step, the PVA is attached to the plate of the FDM equipment and the object is additively manufactured on the PVA where the electronics face off the object (Figure 2b). The object is then detached from the plate of the FDM printer (see in Figure 2c). During this step, the PVA substrate remains firmly stuck to the object because the plastic material that constitutes the additively manufactured object is fused (a highly viscous liquid) when deposited on top of the PVA substrate. Upon drying, there is a stronger affinity between the object and the PVA substrate than between the PVA substrate and the plate of the FDM printer. Note that the temperature of the 3D printer nozzle has been fixed at 215 °C. This temperature is not compatible with the deposition of materials on the PVA substrate. However, the PVA film is not submitted to deformation or other damages that can be induced if the temperature is too high (>160 °C in the case of PVA). It signifies that filament temperature drastically decreases during it deposition (distance between the nozzle and the PVA film). Consequently, the filament deposition temperature has to be chosen according to the PVA requirement and constitutes a limit of this process variant.

The last step consists of dipping the object into the water until the PVA is fully dissolved (see Figure 2d). Figure 2e shows the final result where the PVA is fully removed (i.e., dissolved) and interconnects have been transferred to the object. The optical pictures of Figure 2f show the fabrication steps. The inset in Figure 2f shows the high wrapping capabilities of this process variant. Indeed, even if the 3D object has wrinkles due to FDM manufacturing, interconnects do not show mechanical failure. Moreover, the I(V) curves (see in Figure 2g) highlight an ohmic behavior showing that this technology can be combined with FDM additive manufacturing to add electronics onto a 3D object. Note that the materials and methods could be improved if the 3D-printer was upgraded, allowing the fabrication in the five-axis dimensions. Indeed, in this case, electronics could be attached onto sides or random positions of structures.

However, such a process variant allows us to conformally wrap a reasonably flat surface of a 3D object. Consequently, new technologies have to be investigated in order to wrap a complex surface (sharp edges) of a large-sized object to fit additive manufacturing requirements. The approach consequently benefits from the two previously described techniques. 

#### 3.1.3. Water Transfer Printing (Process Variant 3)

The water transfer printing technology also called hydrographic printing is commonly used in the field of graphic arts to transfer images on the surface of 3D objects [33]. The principle of this technique is to print an image on a hydrosoluble substrate which is then put on top of the water. When the substrate dissolves, the ink floats and remains on the water surface. An object is then slowly dipped through the floating ink, into the water. The film wraps the object and adheres to it. 

Recently, the WTP has been adapted from graphic arts to the field of electronics, allowing the fabrication of passive devices (i.e., Metal/Insulator/Metal capacitors), for instance [26]. As no 2D planar substrate is present during the transfer step, a complex 3D object can be conformally wrapped. In addition to these unique capabilities, the results described in the following will highlight that the water transfer printing technology meets all the requirements to be employed in the additive manufacturing process to embed electronics onto or into 3D objects.

**Figure 3:**
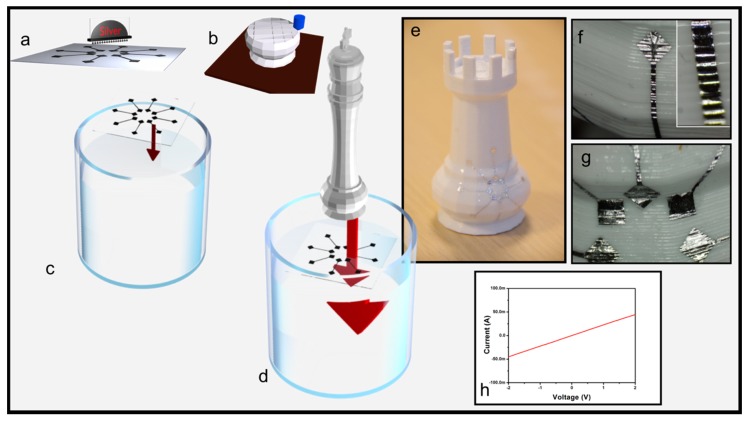
Water Transfer printing technology. 3D schemes showing: (**a**) interconnects printed on water soluble substrate; (**b**) daily life 3D object additively fabricated using FDM; (**c**) PVA substrate deposited on top of water, (**d**) 3D object dipped through floating interconnects; optical pictures showing: (**e**) a chess piece decorated with metallic ribbons arranged in star configurations; (**f**) metallic ribbon wrapping a convex part of the 3D object; (**g**) metallic patterns wrapping a concave part of the 3D object; (**h**) I(V) curve.

The WTP can be considered as another variant of the electronics tattoo process and can be briefly described as following. The electronics are patterned on the PVA substrate first and the structural part can be fabricated in parallel, as shown in Figure 3a,b. After the PVA substrate is placed on top of the water (Figure 3c), the PVA dissolves and the metal pattern floats on the water surface. Then, the 3D object is dipped into the water through the floating pattern and is finally withdrawn (Figure 3c). The thin film pattern wraps the surface as a result of the water pressure, which forces the materials to attach to the object surface (the transfer process is shown in Supplementary Video S2). Optical images in Figure 3d show aluminum transferred onto an additively manufactured 3D object. After the transfer step, the branches of the radial pattern remain unconnected, as shown in the inset of Figure 3e, even if no rigid the substrate is used during the transfer step. Furthermore, the I(V) curve highlights an ohmic behavior, showing no mechanical failure even if patterns are transferred onto sharp edges (bending angle equals approximately 110°). Note that this method does not enable a precise alignment of the electronics with the structural part without any specific apparatus. However, previous work [33] has proposed a solution to overcome this issue by using a camera and a computational tool to allow real-time positioning that could counteract any repulsive forces between the circuit and the part.

### 3.2. PVA Technology Extra-Capabilities

**Figure 4:**
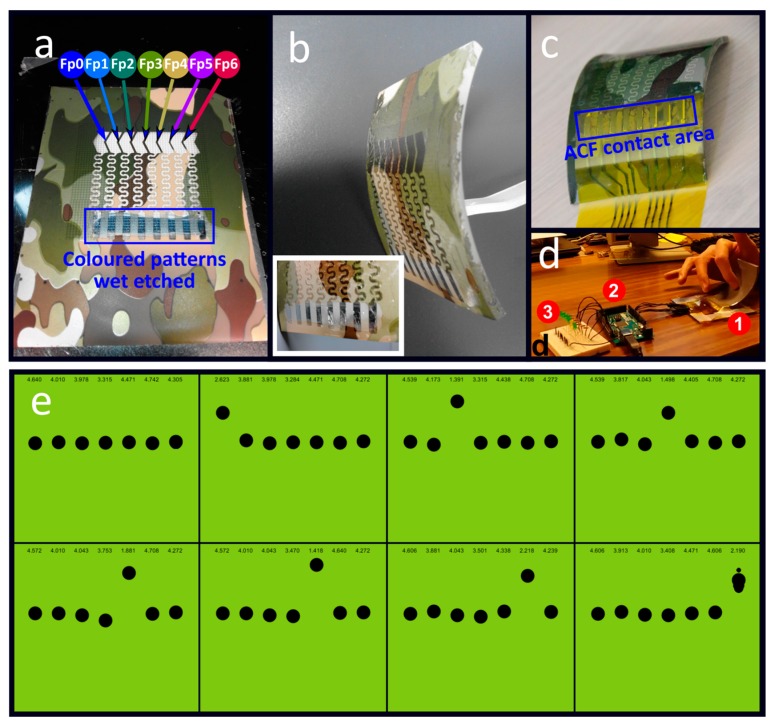
Discrete and hidden electronics applications. Optical picture of capacitive sensors transferred to bent plastic object; (**a**) top-view of metallic electrodes fabricated on colorized PVA substrate. Electrodes constitute the devices sensing area. In blue, the area where part of the colored films has been etched for electrical interconnections access; (**b**) electrodes transferred onto a white object (discrete electronics). The inset highlights a zoom on the area where interconnects can be performed; (**c**) electrodes transferred onto a darker object (hidden electronics) showing electrodes interconnected with the kapton cable using anisotropic conductive film enabling external measurements; (**d**) electrical test setup. The marks 1, 2, and 3 highlight the backside of the capacitive sensors, the Arduino board, and LEDs to display finger displacement, respectively; (**e**) animated graphics showing finger movement tracking using the Arduino board and the Processing software (version 3.4).

As previously highlighted, the WTP shows a better ability to be integrated into the process flow of additive manufacturing. Consequently, the following experiments dealing with the possibility to provide hidden electronics using PVA-based technology have been performed thanks to the WTP concept. Figure 4 shows metallic electrodes fabricated on top of a colorized PVA substrate. The difference between the last set of experiments relies on the colored layer sandwiched in-between the PVA substrate and the electrodes. Optical pictures of the colored PVA substrate and the electrodes are shown in Figure 4a. After the transfer step, the electrodes are in contact with the 3D object and the colored layer covers the electrodes. Consequently, as shown in Figure 4b,c, depending on the colors of the films and the object, discrete (Figure 4b) or even hidden (Figure 4c) electronics can be achieved. However, in order to allow the electrical contact to the electrodes, the colored layer has to be etched (see in Figure 4a) using a solvent such as acetone. Such an etched area is highlighted in blue in Figure 4a.

Each capacitor is highlighted by Fp marks, as shown in (a); (e) back-side of the bent object showing: finger sliding (Fp0 to Fp6) along the sensors area, the Arduino development board, and light emitting diodes showing finger position, marked by the number 1, 2, and 3, respectively.

In order to show applications, the electrodes have been used as capacitive sensors to detect finger positions. Note that the 3D objects’ bending radii equal 5 cm (see in the Figure 4b,c). The sensing area (4 × 1 cm) is defined by eight aligned electrodes (5 mm × 1 cm) in order to detect finger sliding. V-shaped electrodes design is used to optimize the overlap between 1 mm spaced electrodes. Importantly, no mechanical failure was observed, highlighting the robustness of the water transfer printing technology. Moreover, for external acquisition, an anisotropic conductive film (ACF 3M ECATT 9706, 3M, Saint-Paul, MN, USA) was used to connect the capacitive touchpad to a silver ribbon cable printed on Kapton (see in Figure 4c). The capacitive touchpad has been transferred to the convex side of the curved object. As shown in Figure 4d, the finger slides on the opposite side, facing the electrodes, changing the initial capacitance value. Figure 4d shows the Arduino development board (highlighted by the number 2), connected to a computer via serial USB communication, which has been used to measure the capacitance value between each electrode. Capacitances (Cn) are first measured using micro-controller analog outputs/inputs. Time constants (τn) of capacitors discharge τn = R·Cn are then estimated, where R is the pull-up internal resistor of the microcontroller. 

As shown in Figure 4e, when the finger position (Fpn) faces a capacitor (Cn), the capacitance value decreases. Cn values have been recorded and displayed (using Processing software) in numerical values and animated graphics (see in Figure 4e). As expected, Cn is the most impacted by Fpn, enabling finger position sensing. One can note that neighboring capacitors are also, to a lesser extent, impacted by the finger, depending on how it is placed. 

## 4. Conclusions

This works highlights that water-soluble flexible substrates are promising candidates to embed printed electronics devices into structural parts fabricated using additive manufacturing technology. Consequently, fully additive manufactured (structural part and electronics) objects can be achieved that can be a convenient technology to provide low-cost smart objects. Furthermore, among all of the tested process variants, the concept of water transfer printing has shown the best ability to transfer electronics onto complex objects (sharp edge covering and large area processing).

## Supplementary Materials

The following are available online at http://www.mdpi.com/2072-666X/9/9/474/s1. Figure S1: Processing onto PVA substrate using Inkjet printing and conventional photolithography; Video S1: Video showing tattoo electronics process; Video S2: Video Showing Water Transfer Printing Process.
