# Supplementary material for: The Use of a Water Soluble Flexible Substrate to Embed Electronics in Additively Manufactured Objects: From Tattoo to Water Transfer Printed Electronics
Source: Micromachines (Basel). 2018 Sep 17;9(9):474. doi: 10.3390/mi9090474 (PMC6187858; doi:10.3390/mi9090474)
Supplement: Supplementary file 1 [file micromachines-09-00474-s001.zip › micromachines-352738 - for final check - supplementary/supplementary materials/Supplementary materials.docx]

Supplementary Materials

**The Use of a Water Soluble Flexible Substrate to Embed Electronics in Additively Manufactured Objects: From Tattoo to Water Transfer Printed Electronics**

Brice Le Borgne, Emmanuel Jacques and Maxime Harnois

1. Figure

**
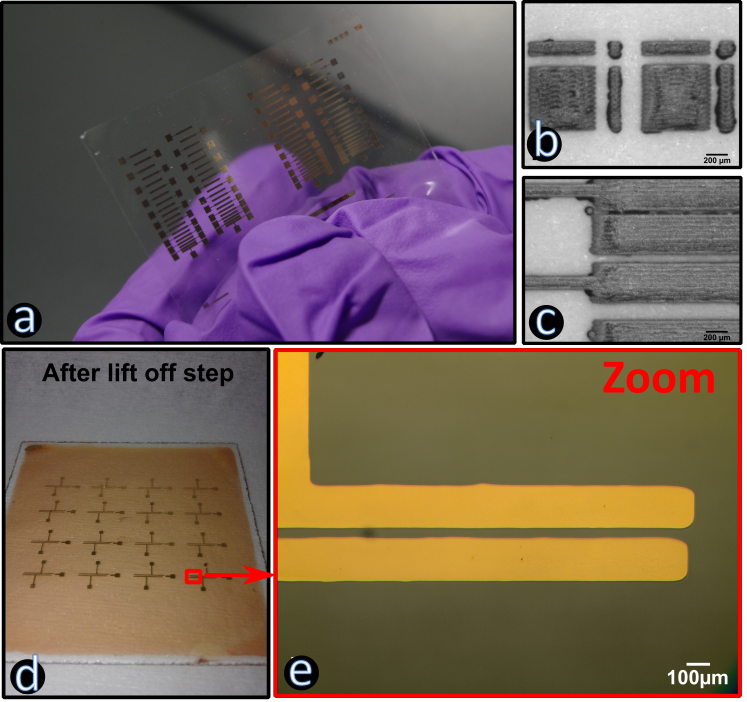
**

**Figure S1.** Processing onto PVA substrate using Inkjet printing and conventional photolithography. (**a**) Identical patterns are designed in columns varying inter-patterns distances (from 40 μm to 1 mm). (**b**) Zoom in top right corner of PVA substrate showing squares (600 × 600μm), horizontal lines, vertical lines and isolated droplets. (**c**) Zoom in patterns with distances are equal to 40 μm, 80 μm and 160 μm (from top to bottom). Due to PVA roughness electrodes spaced from 40μm are connected. (**d**) Thermally evaporated gold electrodes on PVA substrate after lift-off step. (**e**) Zoom on patterns showing the inter-distance electrodes (150 μm width electrodes spaced from 30 µm).

2. Videos

**Video S1:** Video showing tattoo electronics process

**Video S2:** Video showing water transfer printing process
